# The Acute Satellite Cell Response and Skeletal Muscle Hypertrophy following Resistance Training

**DOI:** 10.1371/journal.pone.0109739

**Published:** 2014-10-14

**Authors:** Leeann M. Bellamy, Sophie Joanisse, Amanda Grubb, Cameron J. Mitchell, Bryon R. McKay, Stuart M. Phillips, Steven Baker, Gianni Parise

**Affiliations:** 1 Department of Kinesiology, McMaster University, Hamilton, Ontario, Canada; 2 Department of Medical Physics and Applied Radiation Sciences, McMaster University, Hamilton, Ontario, Canada; 3 Department of Medicine, McMaster University, Hamilton, Ontario, Canada; University of Minnesota Medical School, United States of America

## Abstract

The extent of skeletal muscle hypertrophy in response to resistance training is highly variable in humans. The main objective of this study was to explain the nature of this variability. More specifically, we focused on the myogenic stem cell population, the satellite cell (SC) as a potential mediator of hypertrophy. Twenty-three males (aged 18–35 yrs) participated in 16 wk of progressive, whole body resistance training, resulting in changes of 7.9±1.6% (range of −1.9–24.7%) and 21.0±4.0% (range of −7.0 to 51.7%) in quadriceps volume and myofibre cross-sectional area (CSA), respectively. The SC response to a single bout of resistance exercise (80% 1RM), analyzed via immunofluorescent staining resulted in an expansion of type II fibre associated SC 72 h following exercise (pre: 11.3±0.9; 72 h: 14.8±1.4 SC/type II fibre; p<0.05). Training resulted in an expansion of the SC pool associated with type I (pre: 10.7±1.1; post: 12.1±1.2 SC/type I fibre; p<0.05) and type II fibres (pre: 11.3±0.9; post: 13.0±1.2 SC/type II fibre; p<0.05). Analysis of individual SC responses revealed a correlation between the relative change in type I associated SC 24 to 72 hours following an acute bout of resistance exercise and the percentage increase in quadriceps lean tissue mass assessed by MRI (r^2^ = 0.566, p = 0.012) and the relative change in type II associated SC following 16 weeks of resistance training and the percentage increase in quadriceps lean tissue mass assessed by MRI (r^2^ = 0.493, p = 0.027). Our results suggest that the SC response to resistance exercise is related to the extent of muscular hypertrophy induced by training.

## Introduction

There is a high degree of inter-individual variation in skeletal muscle hypertrophy following resistance exercise training despite exposure to exercise of the same relative intensity [1], [2]. Individuals response to hypertrophic stimuli like resistance exercise leads to a highly variable response with respect to the accretion of lean tissue [1]. The basis for the variability in hypertrophic responses to training is poorly understood; however factors such as genetic variation [3], genetic polymorphisms [4], [5], transcriptomic profiles [6] the ability to activate specific signaling proteins known to be important in muscle protein synthesis [7], and microRNA expression [8] have been identified as potential control points in regulating the hypertrophic response.

Skeletal muscle possesses a functional population of resident stem cells commonly referred to as satellite cells (SC) [9]. SC are activated, proliferate and fuse giving rise to nascent myotubes or fuse to existing muscle fibres in response to various stressors such as mechanical loading or injury [10]. The progression of SC from activation, proliferation through to terminal differentiation is governed by a network of transcription factors referred to as myogenic regulatory factors (MRF) [11]–[13]. Although the essential role that SC play in the maintenance of healthy skeletal muscle function is widely accepted their role in mediating exercise induced skeletal muscle hypertrophy is debatable. Various findings from studies of resistance exercise training-induced hypertrophy in humans highlight the association of SC with muscle hypertrophy [2], [14]. However based on results from rodent models others propose that SC are dispensable in mediating muscle hypertrophy [15], [16]. We propose, however, that because hypertrophy can occur in SC-depleted rodent models does not necessarily render their contribution unimportant in contributing to hypertrophy in humans under physiological conditions. Instead, models of selective SC depletion that demonstrate hypertrophy, under conditions of extreme overload, merely establish the capacity of redundant mechanisms to compensate and result in hypertrophy.

In humans a growing body of evidence has characterized the response of SC to resistance exercise and implied a role for nuclear addition during muscle fibre adaptation [9], [17]–[21]. Only one previous study in humans has, however, attempted to correlate the variability in the myogenic response to acute resistance exercise or following resistance training [2], [14].

Myostatin (MSTN, or growth-differentiation factor 8, GDF-8), a transforming growth factor-beta (TGF-β) family member, is a negative regulator of muscle growth [22]. Knockouts of MSTN in multiple species (including a human case study) resulted in gross muscle hypertrophy and an overall increase in body mass of 2–3 fold as compared to wild-type counterparts [22]. Importantly, there appears to be a conserved role for MSTN in humans, which we demonstrated by showing an acute reduction in the co-localization of MSTN to SC in human skeletal muscle *in vivo* following a single exercise bout. Furthermore, aging was found to be associated with an impaired ability to decrease the proportion of SC co-localized with MSTN following exercise [9]. Collectively, these results imply a role for MSTN in the regulation of human SC function and suggest that MSTN may regulate hypertrophy [23].

The purpose of this investigation was to determine whether either the acute SC response to a bout of resistance exercise or the expansion of the SC pool was related to muscle hypertrophy following training. We hypothesized that individuals with an elevated acute SC response, based on expansion of the SC pool and progression of SCs through the myogenic program, would also demonstrate the greatest increase in lean tissue mass.

## Methods

### Subjects

Twenty-three previously untrained healthy males (age 18–35 years) completed 16 weeks of whole body resistance training. Participants were recreationally active but had no formal weight training experience within the past year. This study was approved by the McMaster University Research Ethics Board and adhered to the principles expressed in the Declaration of Helsinki. Informed written consent was obtained from all participants prior to commencement of the study. Complete subject characteristics have been previously published [24].

### Exercise Training

The training program has been previously described elsewhere [24]. In brief, however, training consisted of four supervised sessions per week, divided into two upper and two lower body sessions. The upper body sessions consisted of chest press, shoulder press, lat pull down, row, bicep curl and triceps extension exercise. The lower body sessions consisted of leg press, leg extension, leg curl, calf press, and abdominal exercise. Training progressed from two sets performed at 70% of 1 repetition maximum (RM) to four sets performed at 85% of 1RM, with all sets performed to the point of momentary muscle exhaustion. At the conclusion of each workout, participants consumed a beverage containing 30 g of whey protein, 25.9 g of carbohydrates and 3.4 g of fat (Musashi p30, Notting Hill Victoria, Australia).

### Acute Exercise Protocol

Prior to the onset of the resistance training program subjects completed an intensity matched (80% 1RM) acute bout of resistance exercise. The acute exercise consisted of four sets and eight reps each of leg press, leg extension, calf press and leg curl. The final set was performed to volitional failure. All exercises (with the exception of leg press) were performed using HUR circuit training-type exercise equipment (HUR, Kokkola Finland).

### Magnetic Resonance Imaging and Dual-Energy X-ray Absorptiometry

Magnetic resonance imaging (MRI) scans of the quadriceps were completed by all participants prior to and following the training program. Prior to scanning, participants rested in the supine position for 1 h to prevent the influence of fluid shift on muscle volume. Imaging was performed in a 3T HD scanner (Signa MRI System, GE Medical, Milwaukee, WI). Image acquisition in the axial plane was performed with the following parameters: repetition time/echo time  = 2100 ms/23.58 ms; field of view  = 28 cm; matrix size  = 320/320 reconstructed to 512/512 phase/frequency; slice thickness  = 5 mm. Thigh image acquisition utilized an eight-channel torso coil with two excitations. There was a 10 mm gap between slices. Quadriceps volume was measured from the first slice where the rectus femoris was visible to the first slice where the gluteus maximus was visible, and calculated by multiplying the slice area by the distance between slices. The area of each slice was determined with Image J software (U. S. National Institutes of Health, Bethesda, Maryland, USA). Time of day, joint angle and leg compression were matched in all pre- and post-training scans.

Body composition was assessed using whole body dual-energy X-ray absorptiometry (DEXA) scan (QDR-4500A, software version 12.31; Hologic, Bedford, MA). Subjects were fasted and had not completed any exercise prior to the scan. All scans were performed and analyzed by a trained technician. All scans were completed according to the AIS whole-body DEXA protocol [24], [25]. Prior to the measurement the DEXA was calibrated as per manufacture guidelines. All subjects were centrally aligned prior to the scan, hands and feet were secured for consistency.

### Sample Collection

Muscle biopsies were obtained from the *vastus lateralis* before, 24 h and 72 h after an acute exercise bout prior to (Pre-1, 24-1, 72-1) and following (Pre-2, 24-2, 72-2) 16 weeks of exercise training using a modified 5 mm Bergström needle with manual suction under local anaesthesia (2% xylocaine). Subjects had not participated in any physical activity at least 96 hours before the collection of the baseline biopsy (Pre). Upon excision, the muscle samples were immediately dissected into pieces that were snap frozen in liquid nitrogen (gene expression analysis), embedded in optimal cutting temperature (OCT) compound for immunofluorescence analysis, or maintained as fresh tissue in growth media (Dulbecco's modified eagle medium - DMEM containing 20% fetal bovine serum - FBS) for flow cytometry analysis.

### Flow Cytometry

Approximately 65 mg of muscle obtained from biopsies prior to training (Pre-1, 24-1, 72-1) were prepared for flow cytometry analysis. Briefly, muscle samples were weighed prior to mulching with sterile surgical scissors in 35 mm tissue culture plates. Single cell suspensions were achieved using enzymatic digestion. 400 µl of Collagenase/dispase solution (10 mg/ml Collagenase B, Roche Diagnotsics, Mannheim, Germany; 2.4 U/ml dispase, Life Technologies, Garlsbad, CA, USA; containing 5 µl/ml of 0.5 M CaCl_2_) was added to each plate, triturated for ∼2 min, and incubated at 37°C for 9 min, this step was then repeated with an incubation time of 5 min. The sample was then filtered using a 70 µm mesh filter and centrifuged at 800 g for 5 min to obtain a pellet of mononuclear cells. Cells were fixed in ice cold 70% ethanol and stored at −20°C. Samples were prepared as previously described [26] by incubation in Pax7 primary antibody (neat, Developmental Studies Hybridoma Bank, Iowa City, IA, USA), Alexa Fluor 488 goat anti-mouse secondary antibody (1∶500, Invitrogen, Carlsbad, CA, USA), and propidium iodide. Flow cytometry analysis was completed on a Beckman-Coulter Epics XL (Beckman-Coulter Inc., Brea, CA, USA) instrument operated by a trained technician.

### Immunofluorescence

Muscle cryosections, 7 µm in thickness, were prepared from OCT embedded samples, allowed to air dry for 15–45 minutes and stored at −80°C. Tissue sections were fixed in 4% paraformaldehyde (PFA) for 10 min, washed 3×5 min in PBST, blocked for 60 min at RT (in PBS containing 2% bovine serum albumin, 5% FBS, 0.2% Triton x-100, 0.1% NaAzide, and 2% goat serum), and subsequently incubated in primary antibodies Pax7 (neat, DSHB), Laminin (1∶250 or 1∶750, Abcam, Cambridge, MA, USA), MHCI (neat, DSHB, Iowa City, IA, USA), MHCII (1∶1000, Abcam, Cambride, MA, USA), and Myostatin (1∶150, Millipore, Etobicoke, ON, Canada) for 2 hr at RT or overnight at 4°C. Secondary antibody detection included Pax7 (Alexa Fluor 594 goat anti-mouse, 1∶500), Laminin (Alexa Fluor 488 goat anti-rabbit, 1∶500), MHCI (Alexa Fluor 488 goat anti-mouse, 1∶500), MHCII and Myostatin (Alexa Fluor goat anti-rabbit, 1∶500), all from Invitrogen, Carlsbad, CA, USA, for 2 hr at RT. Nuclei were labelled with DAPI (4′,6-diamidino-2-phenylindole) (1∶20000, Sigma-Aldrich, Oakville, ON, Canada), prior to cover slipping slides with fluorescent mounting media (DAKO, Burlington, ON, Canada). Images were taken with a Nikon Eclipse 90i microscope at 20× magnification and captured with a high-resolution QImaging fluorescent camera (Nikon Instruments, Melville, NY, USA). SC quantity and myostatin positive cells were quantified using the Nikon NIS Elements AR 3.0 software (Nikon Instruments, Melville, NY, USA) on large scale images consisting of ≥100 fibres/subject/timepoint in a blinded fashion.

### RNA Isolation

RNA was isolated from 15–25 mg of muscle using the Trizol/RNeasy method. All samples were homogenized with 1 mL of Trizol Reagent (Life Technologies, Burlington, ON, Canada), in Lysing Maxtrix D tubes (MP Biomedicals, Solon, OH, USA), with the FastPrep-24 Tissue and Cell Homogenizer (MP Biomedicals, Solon, OH, USA) for a duration of 40 sec at a setting of 6 m/sec. Following a five minute room temperature incubation, homogenized samples were stored at −80°C for one month (samples may be stored up to one year), until further processing. After thawing on ice, 200 µl of cholorform reagent (Sigma-Aldrich, Oakville, ON, Canada) was added to each sample, mixed vigorously for 15 sec, incubated at RT for 5 min, and spun at 12000 g for 10 min at 4°C. The RNA (aqueous) phase was purified using the commercially available E.Z.N.A. Total RNA Kit 1 (Omega Bio-Tek, Norcross, GA, USA) as per manufacturer's instructions. RNA concentration (ng/µl) and purity (260/280) was determined with the Nano-Drop 1000 Spectorophotometer (Thermo Fisher Scientific, Rockville, MD, USA). RNA integrity reported via RNA integrity numbers (RIN scale of 0–10) were determined with the Agilent 2100 Bioanalyzer (Agilent Technologies, Toronto, ON, Canada). Samples were reverse transcribed using the commercially available high capacity cDNA reverse transcription kit (Applied Biosystems, Foster City, CA, USA) in 20 µl reaction volumes, as per manufacturer's instructions, using an Eppendorf Mastercycler epgradient thermal cycler (Eppendorf, Mississauga, ON, Canada) to obtain cDNA for gene expression analysis.

### Quantitative real time RT-PCR

All QPCR reactions were run in duplicate in 25 µl volumes containing RT Sybr Green qPCR Master Mix (Qiagen Sciences, Valencia, CA, USA), prepared with the epMotion 5075 Eppendorf automated pipetting system (Eppendorf, Mississauga, ON, Canada), and carried out using an Eppendorf realplex^2^ Master Cycler epgradientS (Eppendorf, Mississauga, ON, Canada). Expression levels of Pax7 (fwd: 5′-GCTCCGGGGCAGAACTACC-3′; rev: 5′-GCACGCGGCTAATCGAACTC-3′), myostatin (fwd: 5′-TGGTCATGATCTTGCT GTAACCTT-3′; rev: 5′-TGTCTGTTACCTTGACCTCTAAAA-3′), follistatin (fwd: 5′-AGTC CAGTACCAAGGCAGATGT-3′; rev: 5′-GGTCACACAGTAGGCA TTATTGG-3′) and FSTL-1 (fwd: 5′-AGAGGAGGAGATGACCAGATATG-3′; rev: 5′-CGCTGAAGTGGAGAA GATGC-3′) were normalized to the housekeeping gene Beta-2-microglobulin (fwd: 5′-ATGAG TATGCCTGCCGTGTGA-3′; rev: 5′-GGCATCTTCAAACCTCCATG-3′).

### Statistical Analysis

Statistical analysis was performed using Sigma Stat 3.1.0 analysis software (Systat Software, Chicago, IL, USA). One-way repeated measures ANOVA tests, paired two-tailed t-tests and Pearson's correlations were performed where appropriate. Statistical significance was considered to be p≤0.05 with data reported as mean ± standard error of the mean (SEM).

## Results

### Skeletal muscle hypertrophy and nuclear content

**Figure 1:**
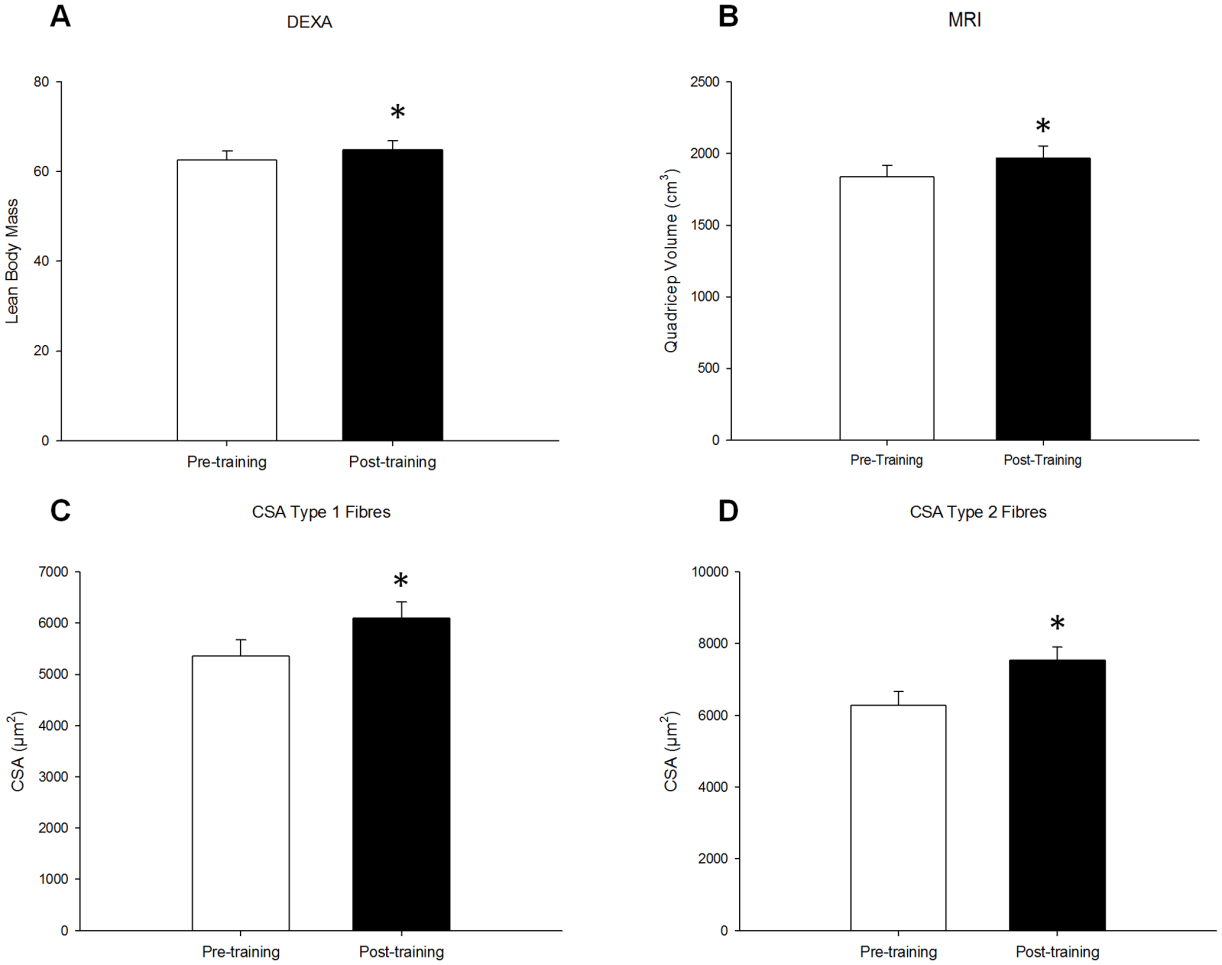
Skeletal muscle hypertrophy following training. Skeletal muscle hypertrophy was assessed by taking measures pre- and post-training using (A) DEXA, (B) MRI, and (C and D) cross-sectional area specific to both type 1 and type 2 fibers respectively. Significant increases were seen in all measures. * denotes a significant difference from pre-training (p<0.001).

**Figure 2:**
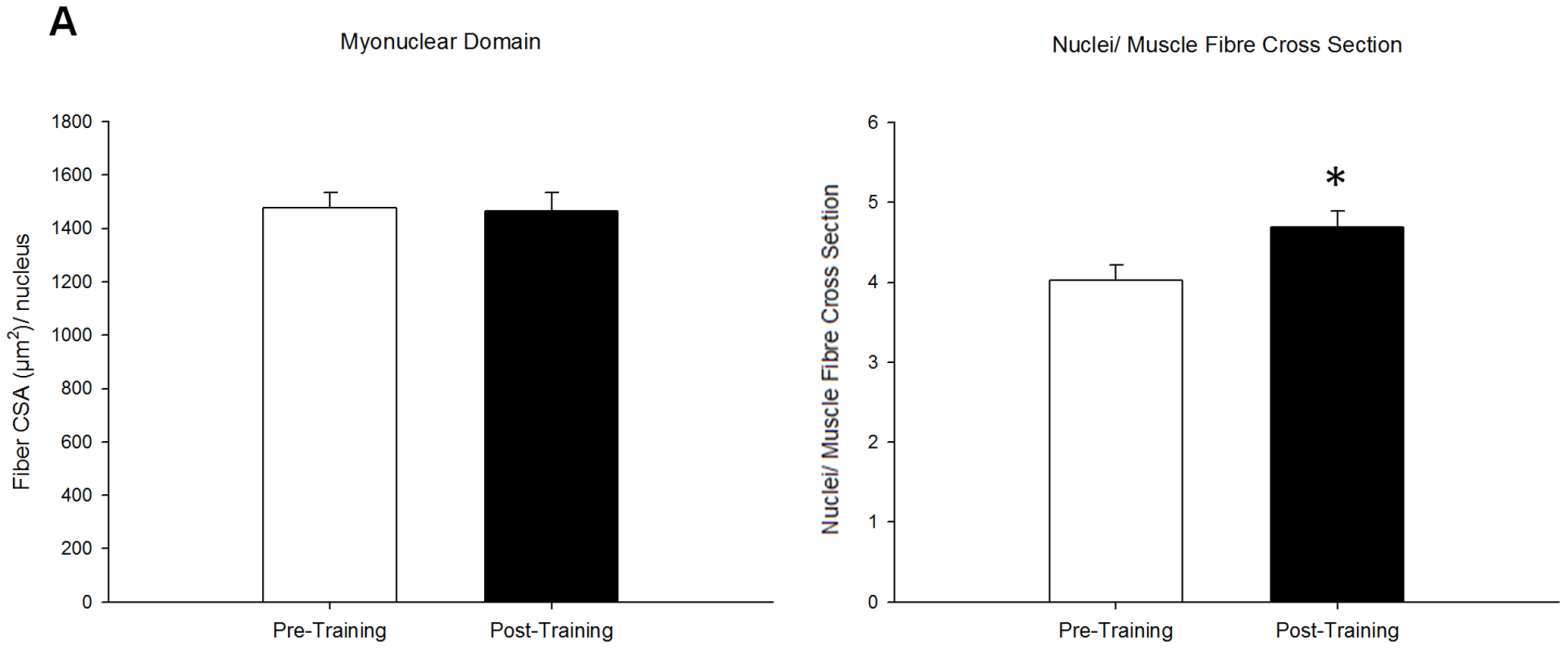
Myonuclear contribution to muscle hypertrophy. Myonuclear content and fibre size were quantified by visualization of nuclei (Pax7/DAPI) and myofibre borders (laminin) on muscle cross sections. Calculations of (A) myonuclear domain (fibre area (µm2) per nucleus) and (B) nuclei per muscle fibre cross section are reported. * denotes a significant difference from pre-training (p<0.05).

Sixteen weeks of whole body resistance training resulted in skeletal muscle hypertrophy as assessed by DEXA, MRI and immunofluorescence (Fig 1). DEXA and MRI scans revealed a significant increase in total lean mass (pre: 62.6±2.0 kg; post: 64.8±2.1 kg; p<0.001) (Fig 1A), and in quadriceps volume (pre: 1837±82 cm^3^; post: 1970±53 cm^3^; p<0.001) (Fig 1B), respectively following training. Fibre CSA was assesed via immunofluorescent staining of muscle cross sections for type I (MHCI) and type II (MHCII); 16 weeks of training increased CSA for both type I (pre: 5355±324 µm^2^; post: 6099±310 µm^2^) and type II fibres (pre: 6284±390 µm^2^; post: 7543±362 µm^2^; p<0.001), as previously reported [24]. Myonuclear domain size, defined as the fiber CSA per nuclei, was maintained in type I (pre: 1409.6±72.5 µm^2^/nuclei; post: 1341.1±61.5 µm^2^/nuclei) and in type II fibres (pre: 1799.6±96.7 µm^2^/nuclei; post: 1987.1±111.8 µm^2^/nuclei), with an increase in the number of nuclei per muscle cross section of type I fibres (pre: 3.9±0.2; post: 4.6±0.2; p<0.05) and a strong trend for an increase in type II fibres (pre: 3.6±0.2; post: 3.9±0.2; p = 0.07) observed with training (Fig 2A and B).

### SC response following acute resistance exercise and training

**Figure 3:**
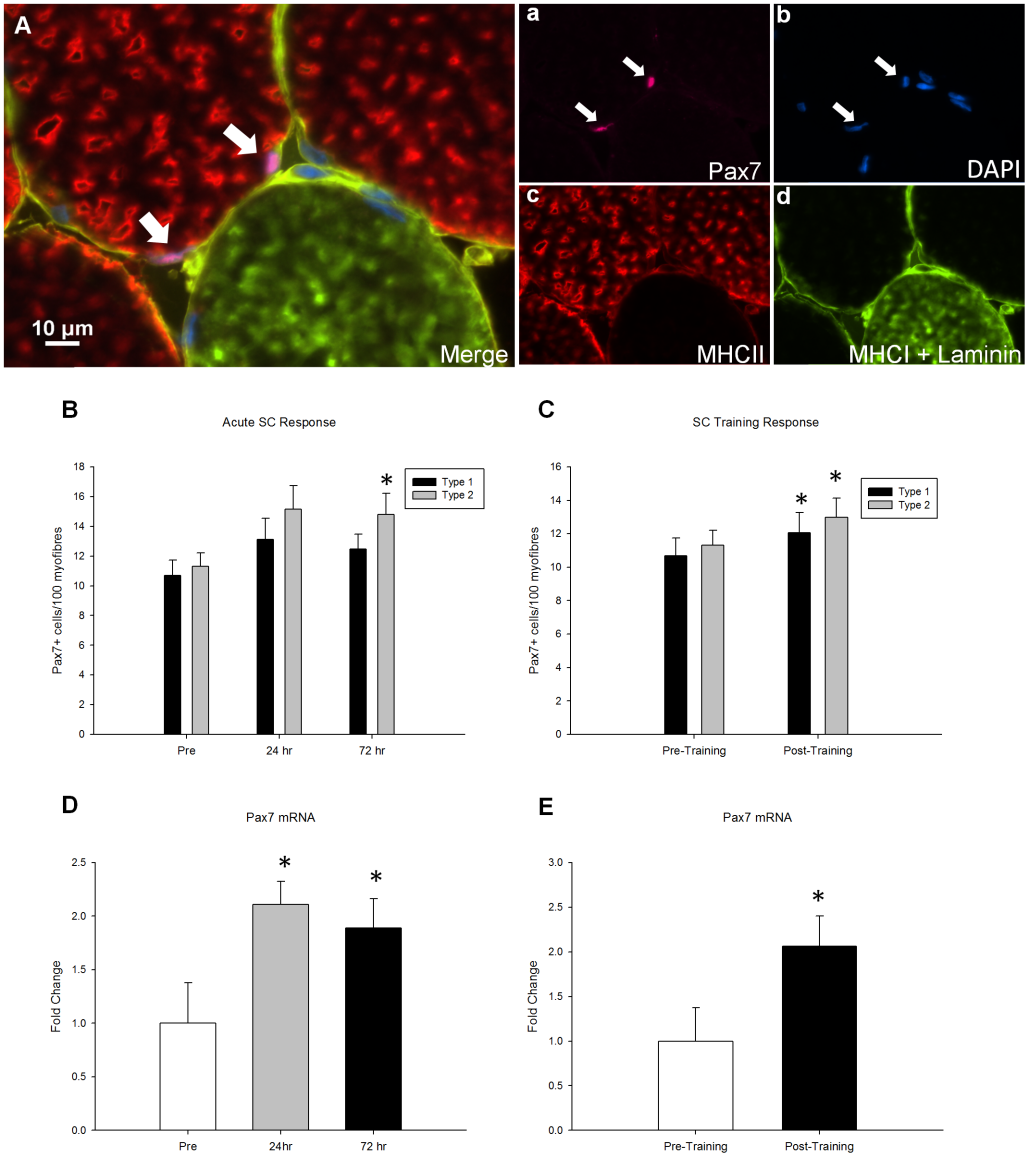
The SC response to an acute bout of resistance exercise and to 16 wks of resistance training. (A), representative image (merge) of a Pax7/Laminin/MHCI/MCHII immunofluorescent stain. Arrow denotes a Pax7+ cell associated with a type 2 fibre. Single channel views of (a) Pax7 (pink), (b) DAPI (blue), (c) MHCII (red), (d) MHCI and laminin (green)are provided. Scale bar measures 10 µm. SC response to (B) an acute bout of resistance exercise and (C) 16 weeks of training are expressed as satellite cell number per 100 myofibres and are specific to fiber type. Changes in SC number correspond to increases in whole muscle Pax7 mRNA in both (C) the acute time course and (D) the training response of Pax7 mRNA. * denotes significant differences from pre- time point (p≤0.05).

The fibre type-specific SC response was quantified via immunofluorescence of muscle cross sections (Fig 3A). 72 hours following an acute bout of resistance exercise the number of SC associated with type II muscle fibres increased (pre: 11.3±0.9; 72 h: 14.8±1.4 SC/100 type II fibres; p<0.05) (Fig 3B). Following 16 weeks of resistance exercise training there was an expansion of the SC pool associated with both type I (pre: 10.7±1.1; post: 12.1±1.2 SC/100 type I fibres; p<0.05) and type II fibres (pre: 11.3±0.9; post: 13.0±1.2 SC/100 type II fibres; p<0.05, Fig 3C).

Gene expression analysis of Pax7 was conducted on whole muscle homogenates. In accordance with the fibre type-specific SC response Pax7 gene expression was increased 2.1-fold and 1.9-fold above baseline at 24 and 72 hours, respectively, following the acute bout of whole body resistance exercise that was completed before the onset of training (p<0.05, Fig 3D). In addition 16 weeks of training led to a 2.1-fold increase in basal Pax7 mRNA expression (p<0.05, Fig 3E).

**Figure 4:**
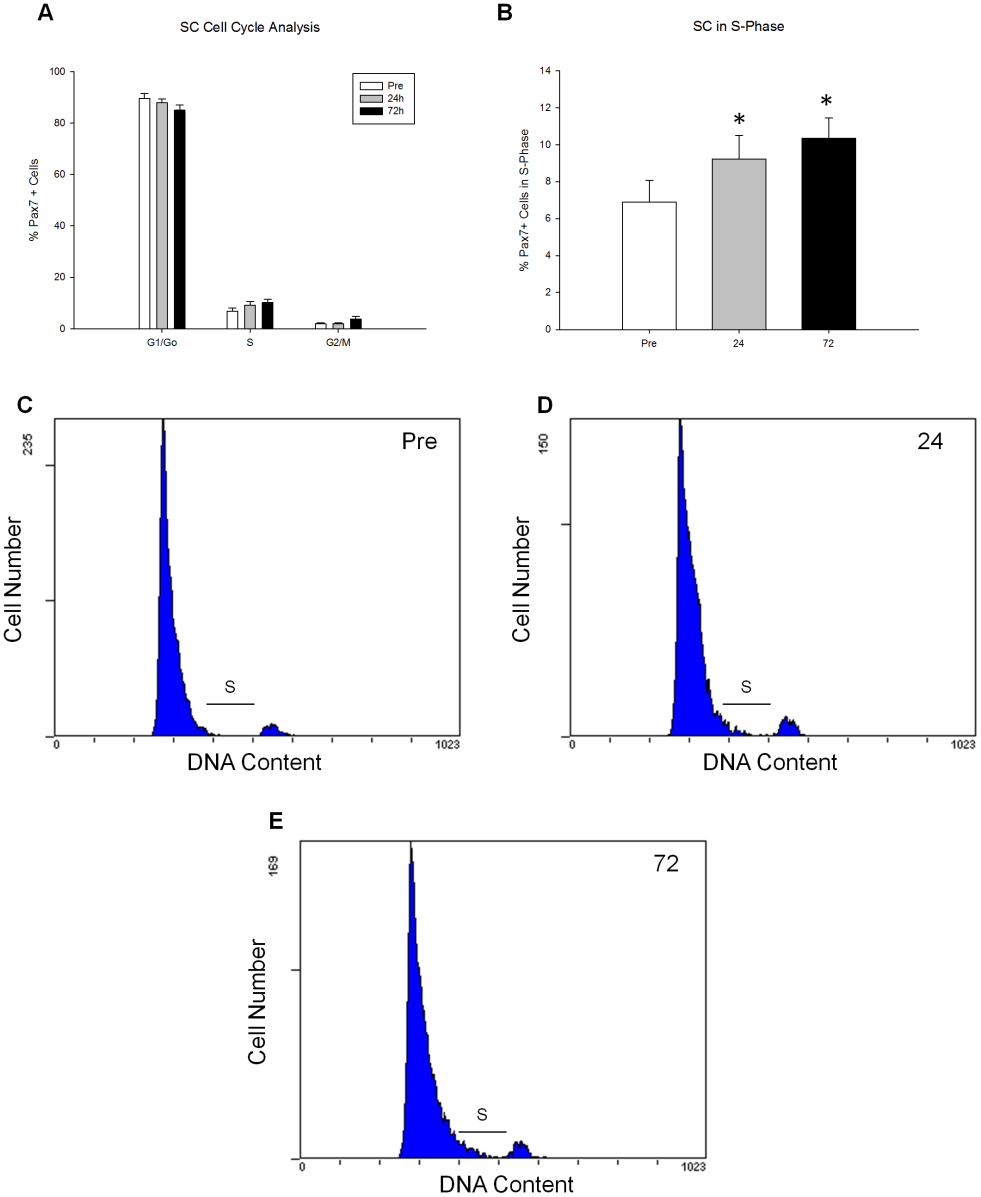
Cell cycle kinetics following an acute bout of resistance exercise. SC cell cycle kinetics in response to a bout of resistance exercise was analyzed through flow cytometry analysis of Pax7/PI staining in the acute time-course pre-training. Percentage of SC (Pax7^+^) in (A) all cell cycle phases (G_o_/G_I_, S, G_2_/M), and (B) S-phase are demonstrated. Representative FACS plots of cell cycle of Pax7+ cells pre (C), 24 h (D) and 72 h (E) following an acute bout of exercise. S phase is highlighted in each representative plot. Pax7+ cells in S phase represent 8.2% (C), 9.2% (D), and 12.3% (E) of total cells pre, 24 h and 72 h respectively. * denotes a significant difference from respective Pre timepoint (p<0.01).

There was an increase in the number of Pax7^+^ cells progressing through the cell cycle as determined by flow cytometry analysis following the bout of acute exercise completed before training. Specifically, the percentage of SC in S-phase progressively increased by 33.9%±14.9 and 50.2%±23.5 24 and 72 hours respectively following exercise (Fig 4). This coincides with the increase in Pax7 mRNA expression 24 and 72 hours post-acute exercise (Fig 3D, E).

### SC response and its relation to muscle hypertrophy

**Figure 5:**
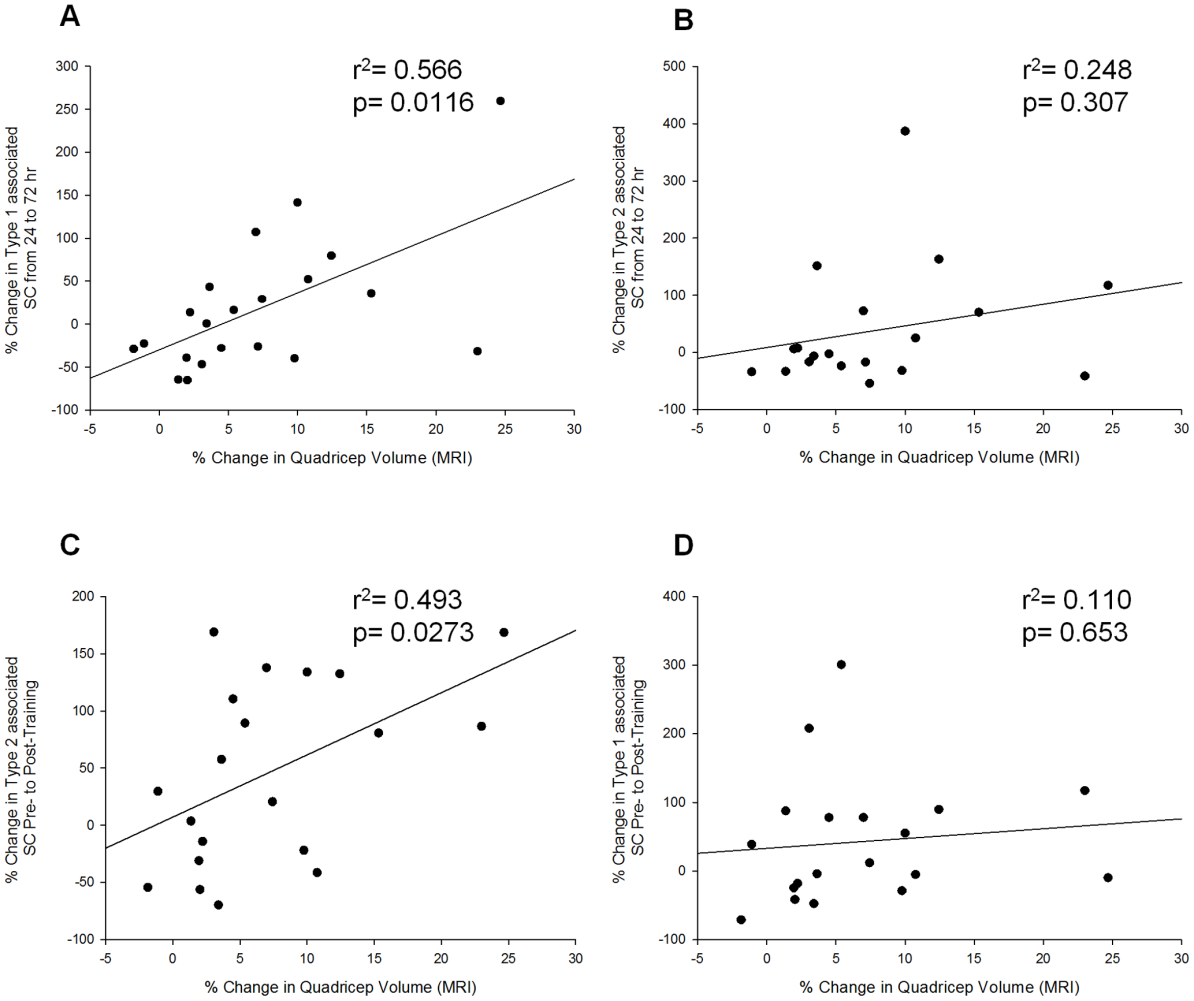
The relationship between the acute SC and teh expansion of the SC pool to skeletal muscle hypertrophy. (A) displays a significant correlation between the increases in quadricep volume as measured by MRI as a result of training and type 1 fibre associated SC pool expansion from 24 to 72 hours after exercise. No such correlation is seen in (B) the type 2 fibre associated SC at that time point. However, a significant correlation (C) was seen between the MRI measured increase in quadricep volume and type 2 fibre associated SC pool expansion as a result of training. No correlation (D) associated with the increase in type 1 fibre associated SC as a result of training was seen with hypertrophy.

To determine the relationship between the acute exercise-mediated SC responses and the changes in baseline SC content following resistance training the relative changes in these variables were individually compared to relative gains in lean tissue mass. Acute exercise completed before training resulted in a highly variable SC response across individuals; however examination of individual responses revealed a significant correlation between the relative changes in SC associated with type I fibres between 24 and 72 hours following acute exercise completed before the onset of training and the relative change in quadriceps volume as assessed by MRI following 16 weeks of training (r^2^ = 0.566; p = 0.012, Fig 5A). There was no correlation in SC associated with type II fibres with the relative change in quadriceps volume following training (r^2^ = 0.248; p = 0.307, Fig 5B). There was a correlation between the change in SC associated with type II fibres following training and the change in quadriceps volume (r^2^ = 0.49; p = 0.027, Fig 5C). However, there was no correlation with the change in SC associated with type I fibres and the change in quadriceps volume following training (r^2^ = 0.110; p = 0.653).

### MSTN co-localization with SC following acute resistance exercise and training

**Figure 6:**
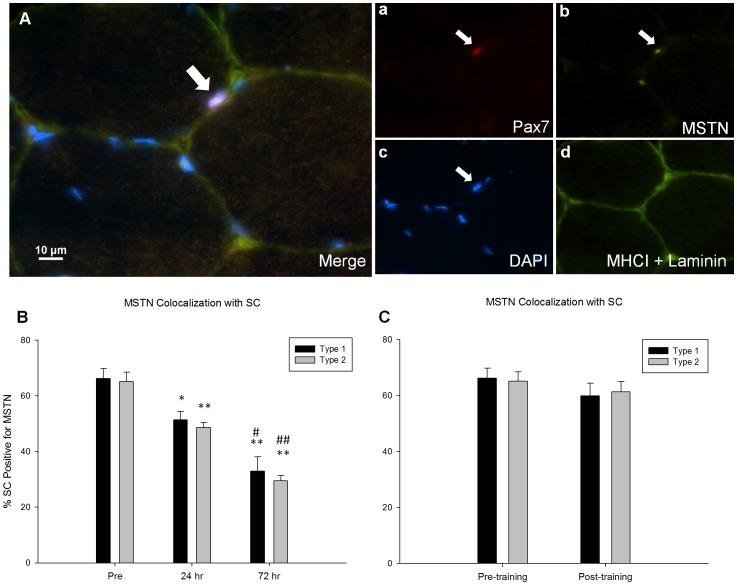
The association of MSTN with SC following an acute bout of resistance exercise and 16 wks of resistance training. (A), representative image (merge) of a MSTN/Pax7/Laminin/MHCI immunofluorescent stain. The arrow denotes a MSTN+/Pax7+ cell associated to a type 2 fibre. Single channel views of (a) Pax7 (red), (b) MSTN (yellow), (c) DAPI (blue), and (d) MHCI and Laminin (green) are provided, scale bar measures 10 µm. Quantification of MSTN colocalization to SCs was done in a fiber type specific manner. The changes in colocalization as a result of (B) an acute bout of resistance exercise and (C) 16 weeks of training is displayed. * and ** denote significant differences from the pre timepoint (p<0.05 and p<0.001 respectively). # and ## denote significant differences from the 24 hour timepoint (p<0.05 and p<0.001 respectively).

To further describe the SC response following acute resistance exercise and following a resistance training program the relation of MSTN, a known negative regulator of myogenesis, specific to the SC was assessed via immunofluorescence of muscle cross sections (Fig 6A). The proportion of MSTN positive SC associated with type I (pre: 66±4%; 24 hrs: 51±3%; 72 hrs: 33±5% type I SC positive for MSTN) and type II fibres (pre: 65±3%; 24 hrs: 49±2%; 72 hrs: 29±2% type II SC positive for MSTN) decreased 24 and 72 hours following acute resistance exercise completed before training (Fig 6B). Resistance training did not affect the proportion of SC positive for MSTN associated with either fibre type (Fig 6C).

## Discussion

For the first time, we report a relationship between the acute temporal SC response to exercise and the accretion of lean mass as a result of exercise training. Together this suggests that the acute SC response is important in the hypertrophic response to resistance training. Additionally, we demonstrated that an expansion of the SC pool following exercise training is also related to muscle hypertrophy. Finally we showed that basal MSTN co-localization to SC following resistance training was not affected with resistance training indicating that basal expression of MSTN within the SC appears to be independent of training status. In agreement with previous, work we also reported an increase in the number of SC associated with type II fibres following acute resistance exercise [9]. This increase in the SC pool following acute exercise was supported by cell cycle analysis demonstrating an increase in the number of SC entering S-phase indicative of SC proliferation to support remodeling. Whether SC are a contributor to muscle hypertrophy has been hotly debated but the question remains unresolved. While ours is not the first investigation to correlate myonuclear content of skeletal muscle cross sections and muscle fibre CSA [1], [27] or demonstrate an increase in SC number in conjunction with hypertrophy [14], [28], [29], we are the first to demonstrate an association between the acute SC response associated with type I fibres following a single bout of resistance exercise with the degree of hypertrophy observed following a resistance training program.

To date, numerous factors have been identified for their role in regulating skeletal muscle hypertrophy yet these factors can only account for a small degree of the variation in muscle growth [30]. During hypertrophy in humans the addition of new nuclei via the SC is necessary in order to support myofiber growth and maintain the myonuclear domain [31]. Consistent with others [1] we observed a great degree of individual variability in muscle growth assessed using various measures. The observation of unaltered myonuclear domain size in both type I and II fibres in addition to an increase in the average number of nuclei per muscle fibre cross section of type I myofibre and a strong trend for an increase per type II myofibre (p = .07) following training strongly suggests a role for nuclear addition in the process of hypertrophy. CSA of type I and II fibres increased following training with a concomitant increase in the number of myonuclei and of SC per fibre suggesting that SC contribute to hypertrophy. Consistent with previous work [27], [32], [33] our data suggests that as myofibre size increases there is a concomitant increase in the progenitor pool to maintain and support growth of the myofibre.

Following acute exercise we demonstrated that the acute response of type I associated SC was associated with skeletal muscle hypertrophy. In line with other reports we observed a significant increase in the number of SC associated with both type II [34] and type I [27] fibres following training, consistent with the increase in CSA observed in both fibre types. Previous work has demonstrated an increase in the basal SC pool following resistance training in individuals who had the greatest gains in muscle mass when ‘clusters’ of responders were examined [35]. Here we extend previous findings showing that the expansion of the SC pool associated with type II, but not type I, fibres was associated with the greatest degree of hypertrophy with training. Collectively, our results along with previous investigations [27], [34] suggest a role for myonuclear contribution in the process of muscle adaptation and growth.

Further analysis of the acute SC response suggests that individuals who saw the greatest gains in muscle mass following training were also the ones with a progressive increase in SC associated, with both type I and II fibres, 24 to 72 hours following acute exercise in all individuals except for one (Figure S2 B, D). While individuals who had the smallest or no gains in muscle mass following training all had a decrease in the number of SC associated with type I and II fibres between 24 and 72 hours except for one individual (Figure S2 A,C). It seems likely that the ability to sustain an increase in the SC response beyond 24 hours following acute resistance exercise is an important factor in determining the degree of muscle hypertrophy. The positive correlation between the number of SC associated with type I fibres from 24 to 72 hours following exercise and the degree of muscle hypertrophy following training suggests that the variability in the response of type I associated SC coincides with gains in muscle mass. All but one of the individuals with the greatest gains in muscle mass had an increase in the number of SC associated with type II fibres following training; while individuals with the smallest/no gains in muscle mass, with the exception of one subject, experienced a decrease or no change in the number of SC associated with type II fibres following training. These data reinforce that although a relationship exists between the acute response of SC associated with type I fibres following acute exercise and muscle growth it is the expansion of the SC pool associated with type II fibres following a period of resistance training that is associated with the greatest gains in muscle mass.

MSTN has been recognized as one of the most potent regulators of muscle mass. Some reports suggest that MSTN gene expression is down-regulated in response to a resistance exercise bout [9], and 24 hours following remobilization after two weeks of immobilization [36]. Furthermore, MSTN has been implicated in the regulation of SC function by impairing activation and differentiation in animals [37] and cell culture models [38]. Recent work from our lab demonstrated an acute down-regulation of the proportion of SC co-localizing with MSTN in response to an acute exercise bout in young and older men [9]. In the current investigation we demonstrate a reduction in the proportion of SC co-localizing with MSTN in both type I and II fibres 24 and 72 hours following acute resistance exercise (Fig 6B); however, following 16 weeks of resistance training with significant hypertrophy, there was no difference in the extent of MSTN co-localization with the SC. This finding is in accordance with some reports which suggest that the inhibition of MSTN resulting in hypertrophy may be associated with changes in MSTN at the myofibre level and not the SC [39], [40]. Although not significant, whole muscle mRNA expression analysis revealed a trend (p = 0.103) for a decrease in MSTN expression following training and a significant increase in follistatin like 1 (FSTL1) expression (Figure S1). FSTL1 is an antagonist of MSTN and its up-regulation may in part mediate the gains in muscle mass following training. Our results suggest that myostatin may play a role in acute SC regulation following exercise but that MSTN-induced training adaptation appear to be mediated at the whole muscle level rather than the SC.

In summary, the results presented in this investigation confirm previous findings demonstrating an acute expansion of the SC pool associated with type II fibres following exercise [9]. Additionally, an expansion of the basal SC pool associated with both fibre types occurred following 16 weeks of resistance training. Although an acute expansion of the SC pool associated with type I fibres was not observed, our study is the first to demonstrate a relationship between the acute response of SC associated with type I fibres and the extent of muscle hypertrophy following training. This highlights that the variability within individuals of the acute type I associated SC response is associated with hypertrophy following training. We also demonstrate a relationship with the expansion of the SC pool associated with type II fibres and the extent of muscle hypertrophy following training. Although MSTN co-localization with SC is down-regulated following acute exercise there is no difference in the proportion of SC expressing MSTN following training. Together these data suggest that under physiological conditions the contribution of nuclei to myofibres by SC is an important event in the regulation of muscle hypertrophy.

## Supporting Information

Figure S1 **Gene expression following 16 wks of resistance training.** The training effect on whole muscle (A) MSTN, (B) follistatin and (C) FSTL1 mRNA. Pre and post- 16 week training measures are shown. * denotes significant differences from p<0.05.
(TIF)

Figure S2 **The acute SC response in the top and bottom 5 gainers.** The type I associated SC response 24 to 72 hours following resistance exercise of the bottom 5 (A) and the top 5 (B) gainers. The type II associated SC response Pre- and Post-training of the bottom 5 (C) and the top 5 (D) gainers.
(TIF)
