# Supplementary figures and images for: The Acute Satellite Cell Response and Skeletal Muscle Hypertrophy following Resistance Training
Source: PLoS One. 2014 Oct 14;9(10):e109739. doi: 10.1371/journal.pone.0109739 (PMC4196938; doi:10.1371/journal.pone.0109739)

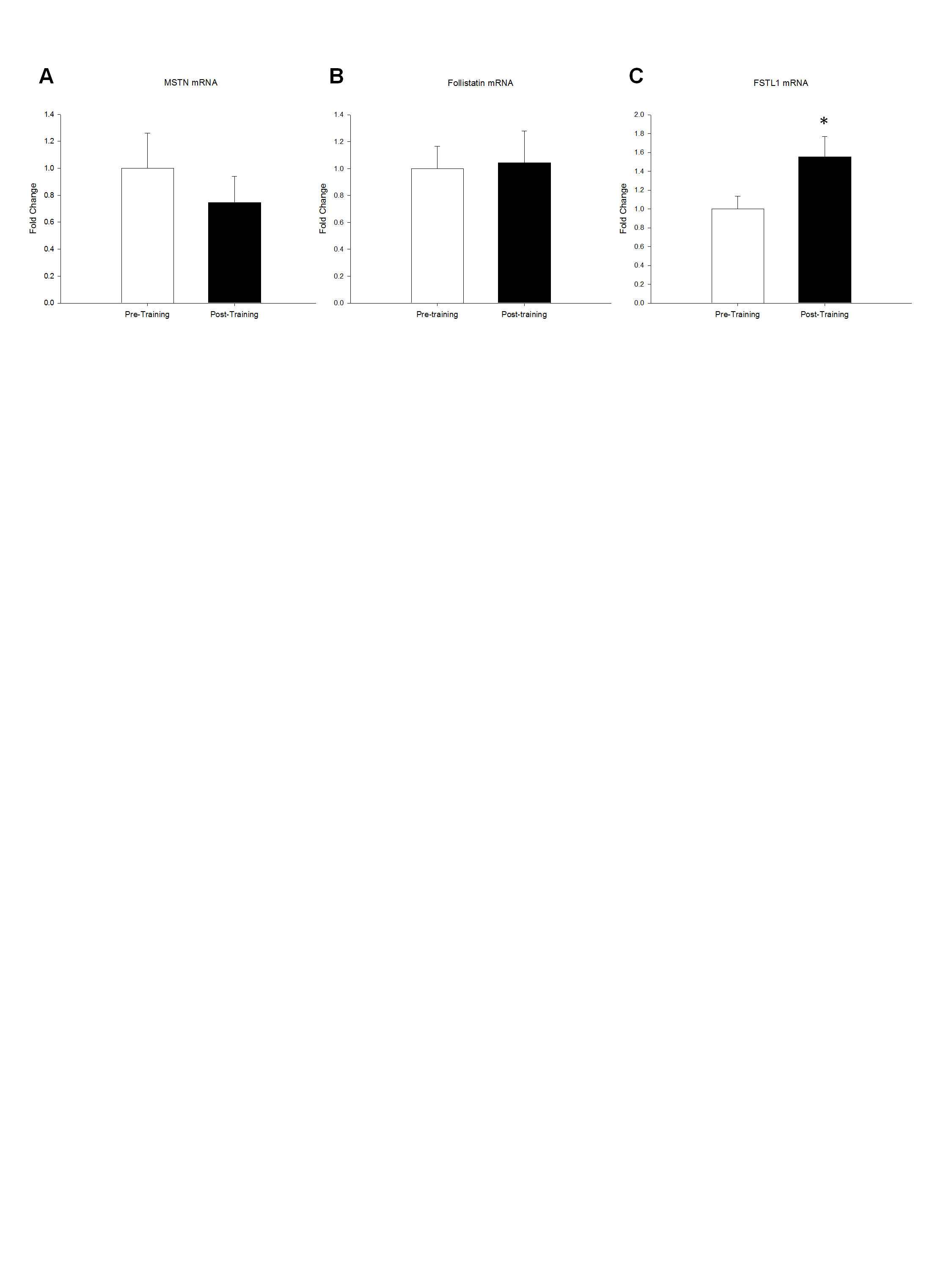

Supplement: Figure S1 — Gene expression following 16 wks of resistance training. The training effect on whole muscle (A) MSTN, (B) follistatin and (C) FSTL1 mRNA. Pre and post- 16 week training measures are shown. * denotes significant differences from p<0.05. (TIF) [file pone.0109739.s001.tif]

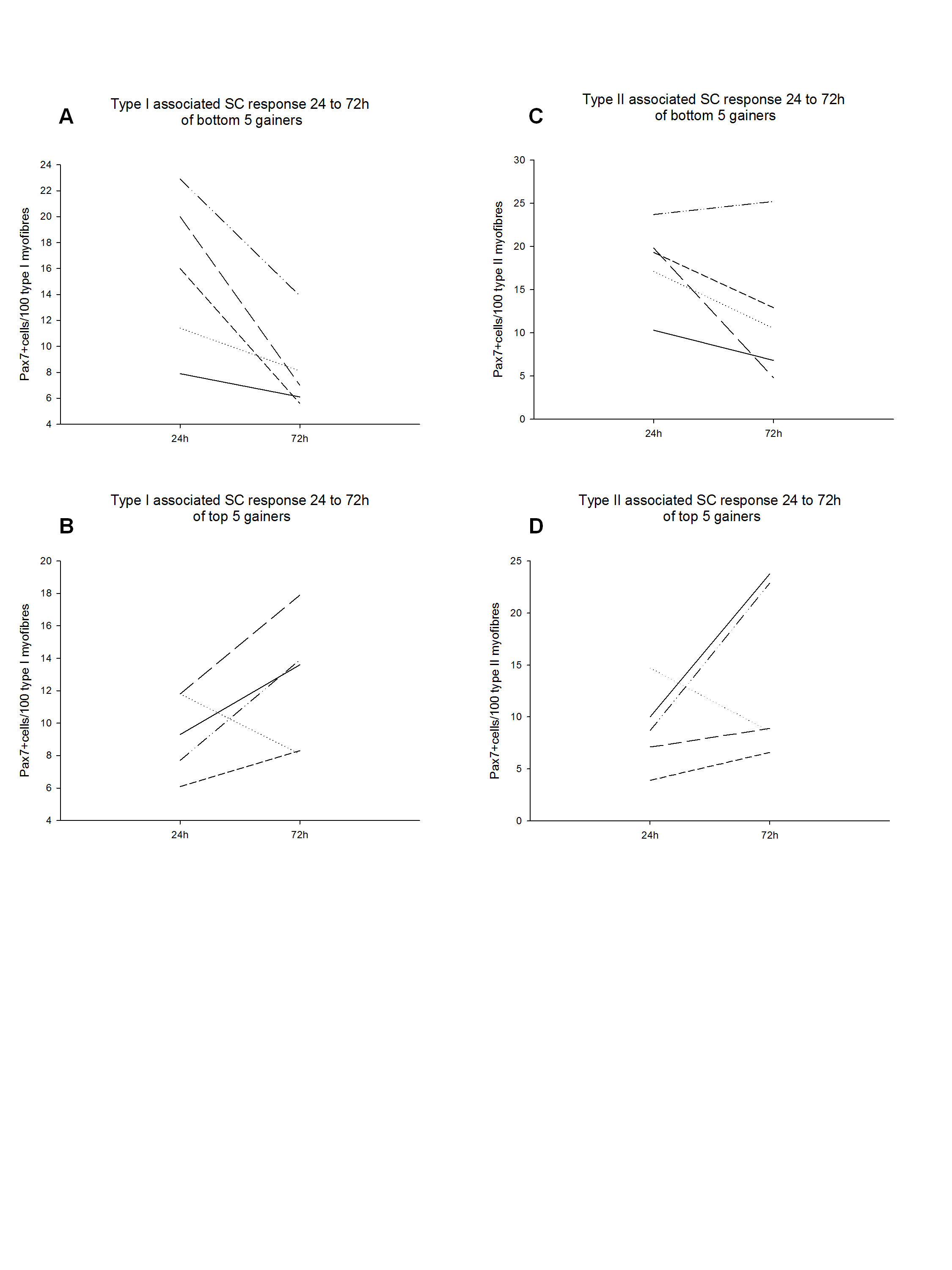

Supplement: Figure S2 — The acute SC response in the top and bottom 5 gainers. The type I associated SC response 24 to 72 hours following resistance exercise of the bottom 5 (A) and the top 5 (B) gainers. The type II associated SC response Pre- and Post-training of the bottom 5 (C) and the top 5 (D) gainers. (TIF) [file pone.0109739.s002.tif]
